# Successful Increase of Outpatient Clinic Continuity in a Fellowship Quality Improvement Project

**DOI:** 10.1097/pq9.0000000000000306

**Published:** 2020-05-20

**Authors:** Ranjini Srinivasan, Peter Sambatakos, Mariellen Lane, Usha Krishnan, Rachel Weller, Jonathan N. Flyer, Keith Robinson, Julie Glickstein

**Affiliations:** From the * Morgan Stanley Children’s Hospital of New York, Columbia University Medical Center, New York, N.Y.; † University of Vermont Children’s Hospital, The Robert Larner, M.D. College of Medicine at The University of Vermont, Burlington, Vt.

## Abstract

**Background:**

Patient-to-physician continuity is the result of coordinated and consistent care. Optimizing continuity can be a challenge in medical training without impacting work hours. We sought to use quality improvement science during graduate medical training to increase outpatient continuity.

**Objective:**

The primary goal was to improve outpatient continuity in our pediatric cardiology fellowship, without increasing trainee clinic hours, from a baseline of 38% to ≥70% within 18 months.

**Methods:**

Our fellowship conducted a quality improvement project across 3 years to improve continuity-of-care in our outpatient clinic using the Institute for Healthcare Improvement model for improvement. We conducted Plan-Do-Study-Act cycles and completed a key driver diagram using a multidisciplinary team. We defined continuity as a patient being evaluated by their primary fellow or a different fellow that was provided a handoff. The outcome measure was the continuity rate over 2-week periods.

**Results:**

Continuity improved from 38% to ≥80%. The improvement resulted from a series of interventions, including creating a handoff system among fellows, identifying follow-up patients in advance, and communicating this information to the clinic team. Although we anticipated a decrease when new fellows were incorporated, continuity continued to be ≥70%. This system retained continuity above 90% one year after completion of the project.

**Conclusions:**

Our fellowship created a system change to improve primary patient-to-fellow continuity care rates. We achieved sustainable continuity by working with a multidisciplinary team without altering staffing, infrastructure, or fellow work hours. This project engaged trainees to address the practical application of quality improvement methodology to solve a common clinical problem.

## Abstract 2

Supplemental Digital Content is available in the text.

## INTRODUCTION

Continuity of patient care is defined as an ongoing medical relationship between physician and patient with participation and cooperation from the healthcare team.^1^ It may be further divided into 3 categories: informational, management, and relational.^2^ Informational continuity includes the transfer of information, management involves uniform care, and relational pertains to the therapeutic physician–patient relationship.^3^ All 3 forms of continuity create a cohesive experience for the patient and allow providers to focus on the care of the individual while promoting quality health education and management.^4^ Improving outpatient continuity may result in fewer hospital admissions, shorter length of stay, and decreased health care resource utilization.^5,6^ Consistent patient and care team relationships may improve patient satisfaction, including the perception of provider medical knowledge, thoroughness, and interest in patient education.^7^

Graduate medical programs are required to show evidence of patient continuity to meet current accreditation standards. However, this process is not well defined.^8^ Continuity-of-care between trainee and patients appears to improve clinical outcomes.^9^ Also, promoting continuity-of-care increases trainee knowledge of a longitudinal disease, emphasizes the importance of professionalism, and creates more meaningful patient–trainee relationships.^8–10^ Other pediatric training programs have tackled improving continuity by increasing time spent in the clinic, with improvement in continuity rates.^10^ Although increasing time in the clinic may help achieve these goals, this is not always feasible in a clinically demanding training program, where trainees must balance education with the rigors of clinical responsibilities.^11^

Although continuity is essential for both quality clinical care and education, to date, no medical training program has published quality care metrics demonstrating baseline metrics and improvement in its outpatient continuity rates of fellowship physicians. Based on informal feedback, graduating fellows from our program recognized a need to improve outpatient continuity. Ongoing concerns included that the outpatient clinic experience was “not time-efficient” and “frustrating.” Fellows saw a mix of new patients and follow-up patients previously evaluated by another fellow. There was no formal clinical care handoff. Providing care to another trainee’s primary patient required additional time to review prior documentation and striving to establish a new physician–patient bond. Based on this feedback, this project sought to investigate and address patient continuity rates in a busy, urban, and academic setting through quality improvement (QI) science. The SMART (specific, measurable, actionable, realistic, and timely) aim of this fellow-led QI project was to increase the pediatric cardiology fellowship outpatient continuity, without increasing trainee clinic hours, from a baseline of 30% to ≥70% within 18 months. The primary goal was to increase informational continuity, with secondary goals of improving management and relational continuity.

## METHODS

Our fellowship program included 15 fellows. Typically 5 of the 15 fellows were responsible for a weekly, full-day outpatient clinic with ≈700 patient visits per year. The fellows assigned to the clinic each week varied based on their rotation schedule. Trainees were supervised by 3 senior faculty. The fellows present in the clinic changed from week to week, but the attendings remained the same. Support staff included 2 nurses and 2 administrative assistants, who were able to schedule follow-up appointments at the end of the visit or over the phone. The time slots were 1 hour for new patients and 30 minutes for follow-up patients. The pediatric patients in our clinic ranged from neonates to 20 years old.

**Fig. 1.**
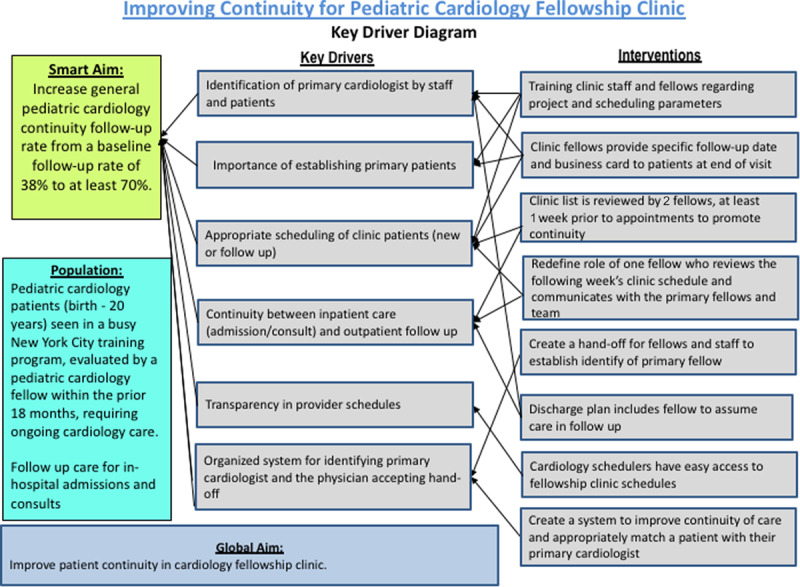
Key driver diagram (KDD).

Primary data collection took place from March 2015 to March 2017, with additional data collected 1 year later to assess sustainability. At the initiation of our project, there was a multidisciplinary meeting to understand the current workflow and identify potential obstacles. This meeting included the entire multidisciplinary team: all 15 fellows, clinic nurses, administrative assistants, and clinic faculty. Based on this feedback, we developed a key driver diagram (Fig. 1) to identify barriers and outline Plan-Do-Study-Act (PDSA) cycles.^12^ We used the key driver diagram to plan interventions. We identified several themes when assessing the barriers to continuity: lack of transparency in the fellows’ outpatient schedule, inability to determine the primary fellow without reading previous notes, lack of follow-up patients in the clinic, and poor communication between clinic staff and fellows. The administrative assistants voiced that the lack of communication led to confusion regarding when and with whom to schedule patients.

We used the SQUIRE (Standards for Quality Improvement Reporting Excellence) 2.0 guidelines to report our findings.^13^ Two senior fellows coordinated the project but incorporated the entire multidisciplinary team. We did not collect patient demographics or clinical information and were exempt from the Columbia University Institutional Review Board process.

The primary fellow was defined as the fellow who had previously evaluated the patient in the outpatient or inpatient setting. Our operational definition of continuity was matching the patient with either the primary fellow or a separate fellow assigned to the clinic that day who was provided a clinical handoff. The clinical handoff was given before the start of the clinic and included relevant patient history and an up-to-date assessment of the patient’s clinical care plan. The handoff provided more information than merely reading the patient chart because it conveyed the thought process of the fellow in managing that patient and clear goals for the visit. The handoff also allowed the primary fellow to remain connected to their patient’s care, even if they were unable to evaluate the patient physically in the clinic. We defined continuity rate as those patients who fulfilled our continuity criteria divided by the total number of patients seen in the clinic that day. The outcome measure was the continuity rate over a 2-week period.

**Fig. 2.**
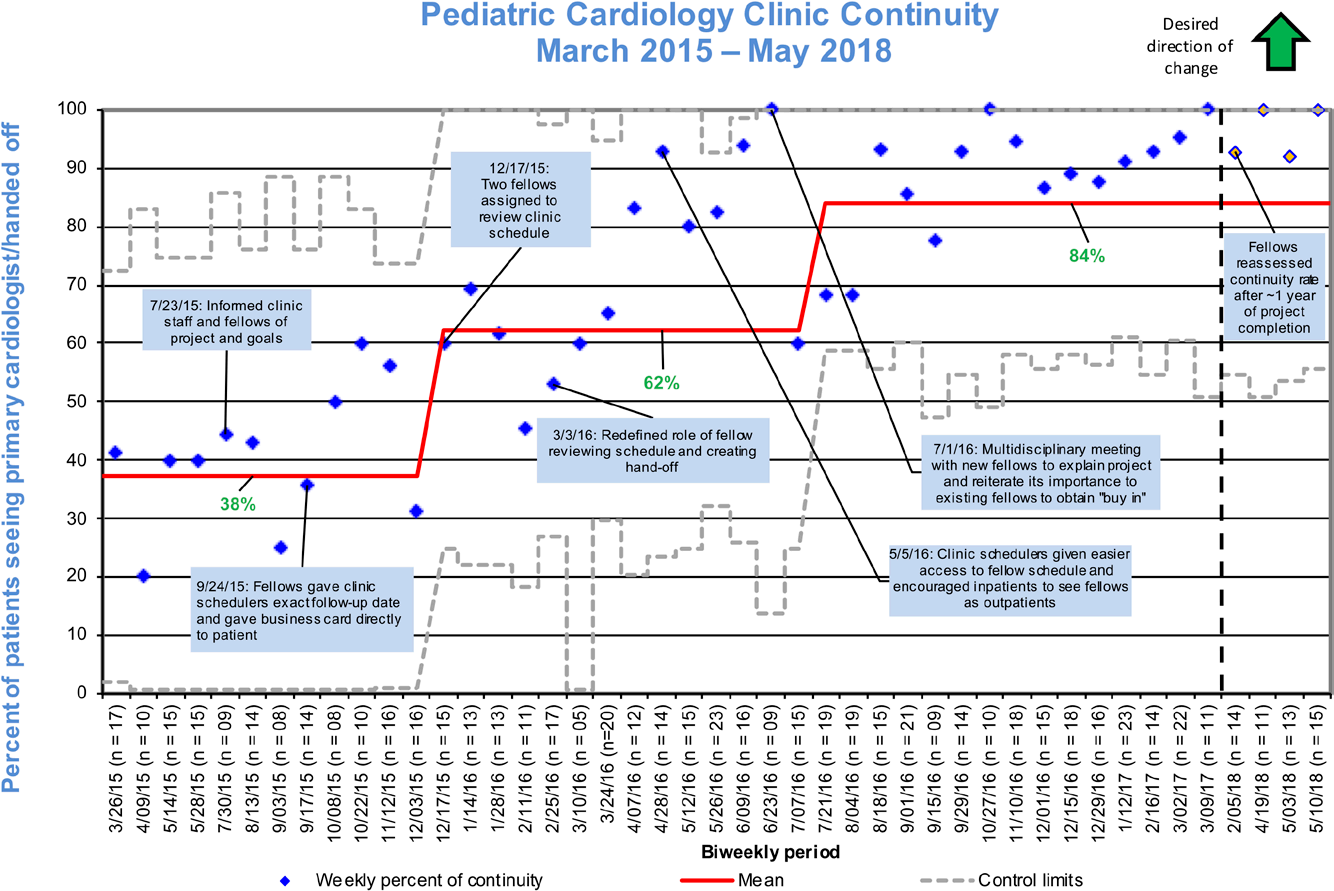
Process control chart with bimonthly follow-up patients and percent of patients seeing the appropriate primary cardiologist.

A statistical process control chart studies how a process changes over time.^14^ We analyzed our data using this chart (Fig. 2) on a biweekly basis to ensure that the denominator was >5 per evaluation period. We excluded patients seen >18 months prior due to the higher likelihood of the primary fellow having graduated. We also analyzed data for special cause variation, as determined by at least 8 data points above or below the centerline.^15^

All of the interventions were guided based on input from the multidisciplinary team. Each PDSA cycle was 2–3 months. After each cycle, we met to discuss prior results and potential future interventions. The initial multidisciplinary meeting highlighted the need to improve communication between physicians, patients, and clinic staff. Therefore, our first intervention identified primary fellows and fellows provided their business cards to the family. The primary fellow then provided an exact follow-up date at the end of the visit.

In the next meeting, several fellows suggested that taking on an active role in scheduling might alleviate some of the continuity conflicts. The next key intervention created a 2-fellow team to review patients scheduled for the upcoming week and to determine if the patient was appropriately scheduled with their primary provider. At this time, 2 fellows created a list that identified each patient and their primary fellow. We circulated this list to the clinic staff, schedulers, and fellows.

In the next intervention cycle, the 2-fellow team was changed to a single fellow, and the patient list became a formal, written handoff, which was created a week in advance of the clinic. The fellow reviewed the schedule for the following week, identified established patients based on past notes, and created a list of follow-up patients. If the primary fellow was not scheduled to be in the clinic, that individual was notified. It was incumbent on the primary fellow to provide written pertinent clinical information about the patient, tests to be ordered, and planned goals for a visit, which was incorporated into the handoff. It was also the responsibility of the primary fellow to identify the co-fellow who would evaluate the patient.

Next, both faculty and fellows proposed that a department-wide change might increase the number of follow-up patients in the clinic. This proposal led to a critical intervention to change the referral process to encourage inpatients to follow-up with the fellow who performed their initial inpatient consultation. The final intervention was to have increased transparency in scheduling by giving clinic staff the fellows’ clinic schedule for the entire year.

As a balancing measure, we retrospectively surveyed the fellows anonymously about their satisfaction with the outpatient clinic experience at the end of 18 months. The goal was to ensure that the additional work and time required on the part of the fellows were not so difficult that it negatively impacted the fellows’ attitude toward the outpatient experience. Fellows rated satisfaction on a 5-point Likert scale from 1 (very dissatisfied) to 5 (very satisfied) (see figure, Supplemental Digital Content, available at http://links.lww.com/PQ9/A189).

## RESULTS

Baseline data were obtained from March to July 2015, with mean continuity of 38% (Fig. 2). Approximately 20–25 patients were scheduled each week, and nearly 40% were follow-up patients. Each intervention cycle was approximately 2–3 months long.

There was special cause variation after December 2015 and July 2016. The first episode of special cause variation shifting the mean from 38% to 62% was seen in December 2015 following 2 interventions: initial project education and encouraging fellow to engage clinic schedulers and patients. In July 2016, there was special cause shifting of the mean to 84% after 2 fellows were assigned to review the clinic schedule. Other interventions in this period included redefining the fellow’s role in creating a handoff, improved access to the clinic schedule for staff, and education of new fellows.

When the senior fellows managing the project graduated, they handed over the responsibility of the project to 2 first-year fellows. Despite the loss of graduating fellows and incorporation of new fellows in July 2016, the continuity remained above the goal of 70%. Over this period, the mean continuity rate remained at 84% without special cause variation. At multiple survey points, 1 year after this transition, the continuity remained above 90%. A small follow-up sample of 4 data points collected in 2018 suggested the mean continuity rate remained above our goal.

**Fig. 3.**
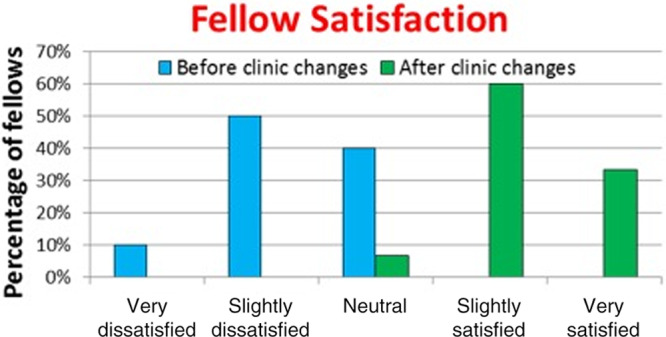
Responses from the fellowship survey after 18 months of PDSA cycles (N = 15).

The results of the retrospective survey are included (see figure, Supplemental Digital Content, available at http://links.lww.com/PQ9/A189). At the outset, none of the fellows reported satisfaction with their outpatient experience. At the end of the project, 93% of fellows reported being satisfied. Fifty-three percent of fellows were seeing their primary patients “quite often” (Fig. 3). This balancing measure showed that the fellows did not find the project to be burdensome, but rather, found increased satisfaction in the continuity clinic.

## DISCUSSION

Our primary goal was to increase informational continuity in a medical training program to create a system where past information was used to make appropriate decisions to manage each patient’s health condition. The secondary goal was to increase management and relational continuity by creating seamless transitions in care while maintaining a therapeutic relationship between patient and provider. This goal was accomplished through a fellow-led QI project to address the challenge of continuity-of-care in a trainee outpatient clinic. Continuity increased to ≥70% within 18 months through a series of interventions, most successful of which was reviewing the schedule in advance, creating a structured handoff, and creating an open dialogue with a multidisciplinary team. These interventions improved communication within the healthcare team, allowing smooth transitions in providing quality outpatient care. This system remains in place and appears to be performing at a very high level a year later.

We used informational continuity in creating a fellow handoff. The handoff provided the interim physician with knowledge of the patient’s medical history and the purpose of the visit. While the ideal circumstance would include the same fellow–patient dyad at all times, in a clinically demanding program, this is not always possible. The Accreditation Council for Graduate Medical Education (ACGME) documents handoffs as an intricate part of trainee education to maintain continuity in the duty-hour restricted era.^16,17^ In a busy training program, the handoff minimized interruption in care for the family and assisted fellows in adhering to training work hour guidelines and competency requirements. We addressed management and relational continuity by increasing the frequency that the original fellow followed up with their patients and created consistent care plans.

A prior study demonstrated increasing pediatric trainee time in the clinic as a means of improving continuity.^18^ Our project improved continuity without changing the time spent in the clinic. There is a published concern regarding pediatric cardiology training that outpatient care has been neglected and that fellows are more comfortable with ICU management.^19^ We addressed this concern by involving the entire fellowship in outpatient care, which placed greater emphasis on its importance and, based on the retrospective survey, contributed to fellows feeling increased ownership of their clinic patients.

Program leadership initiated this project, although it was led by trainees to address significant patient care and system issues. Fellows collected baseline continuity data and led a multidisciplinary improvement team in designing and implementing interventions. We intermittently presented performance data to the fellowship and reviewed it as part of an ongoing QI training process. The ACGME stresses QI activities in “developing the ability to identify and institute sustainable systems-based changes to improve patient care.” The trainees changed the system in which they worked and shifted the culture toward the delivery of quality care within a healthcare team. This change also stressed the importance of longitudinal care and responsibility in managing primary patients.

Our continuity-of-care workflow model also used a team approach with appropriate communication between its members. This model attempts to shift the current continuity culture from the patient–physician relationship to a collaborative team approach.^20^ We addressed interpersonal and communication skills by changing the dialogue between physicians, nurses, and administrative staff. All team members were invited to PDSA meetings and were diligent in attending. After several cycles, the staff expected to receive a clinic list identifying patients and their respective fellow each week.

There were some limitations to this project. We did not survey the patients, which is a shortcoming in evaluating how our project impacted patient experience, and this information may have guided our interventions. A patient survey may have allowed us to assess for continuity based on the patient’s perspective. Generalizability is a potential limitation as our clinic does not pair fellows one-on-one with an attending, as may occur in other programs. Another limitation was the time required for fellows to review the clinic schedule. This limitation could be addressed by using support staff to review the weekly schedule, given that they were already engaged in the QI project. Finally, there was potential lag time bias in our earlier interventions, given that the follow-up interval varied from patient to patient. This delay makes it difficult to determine which interventions were directly responsible for special cause variation. This issue may be addressed in the future by removing specific interventions and studying the continuity rate.

Future directions may include a patient/family advocate on the multidisciplinary team and using the electronic medical record to identify the primary cardiologist. This project may be expanded to other subspecialties clinics to determine if it is generalizable beyond pediatric cardiology. A long-term goal may include the effect on healthcare utilization to determine if increased continuity correlates with fewer tests ordered.

This ongoing QI project successfully addressed the deficit of continuity of care in our outpatient clinic. The program created a culture of multidisciplinary communication without significantly altering the structure of the clinic or increasing trainee outpatient hours. Ultimately, this change educated fellows on QI science and may have established stronger bonds with their patients. This QI project demonstrated sustained improvement 1 year postimplementation, with changes that have become ingrained in the culture of this outpatient clinic and may be adapted to other medical training programs.

## DISCLOSURE

The authors have no financial interest to declare in relation to the content of this article.

## ACKNOWLEDGMENTS

We gratefully acknowledge the pediatric cardiology fellows from Morgan Stanley Children’s Hospital of New York.

## Supplementary Material


