# Supplementary material for: Successful Increase of Outpatient Clinic Continuity in a Fellowship Quality Improvement Project
Source: Pediatr Qual Saf. 2020 May 20;5(3):e306. doi: 10.1097/pq9.0000000000000306 (PMC7297393; doi:10.1097/pq9.0000000000000306)

Supplemental Material:

Survey sent to all fellows at the completion of the project. All 15 fellows responded.


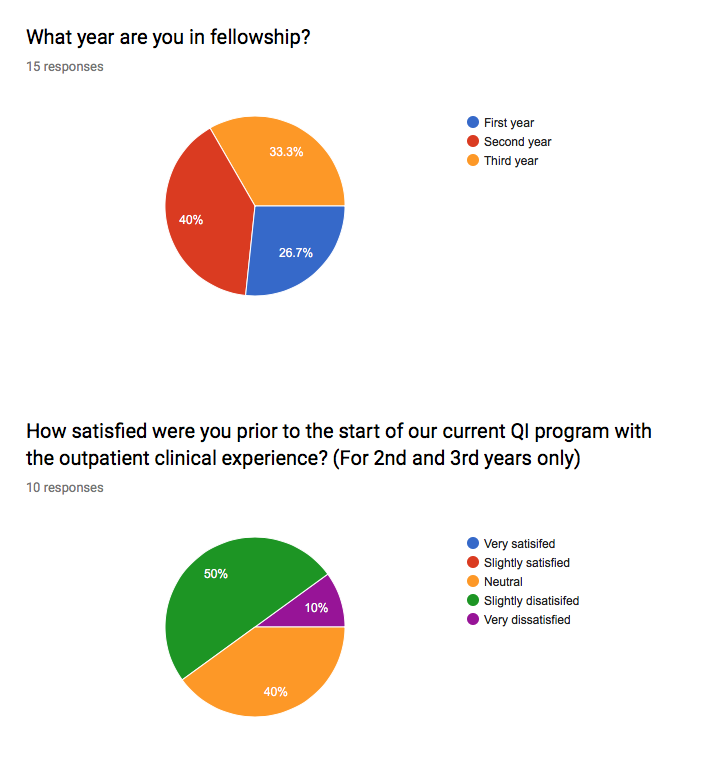


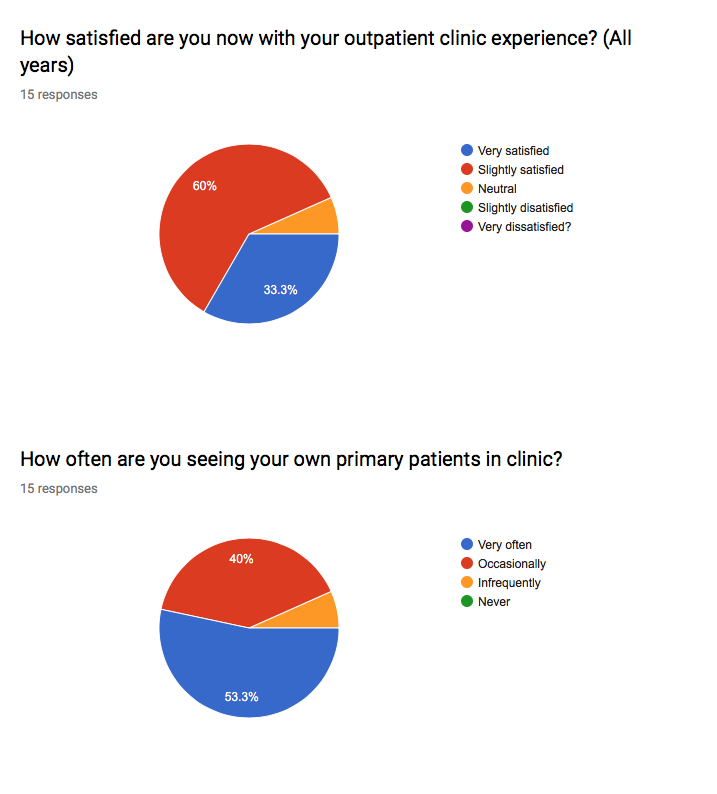


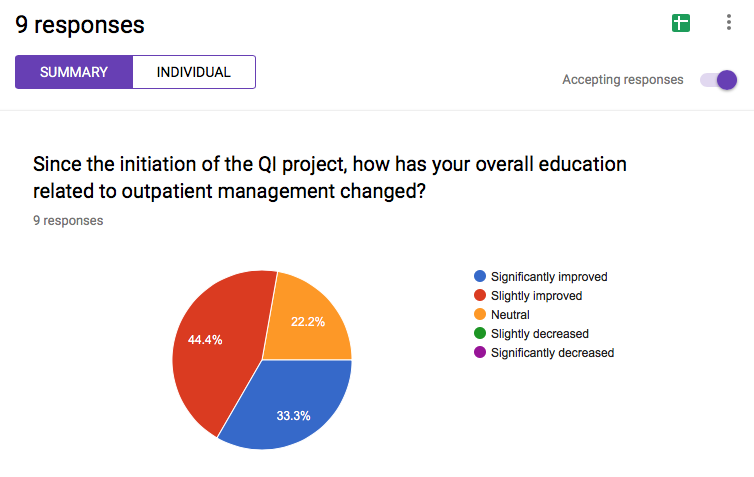


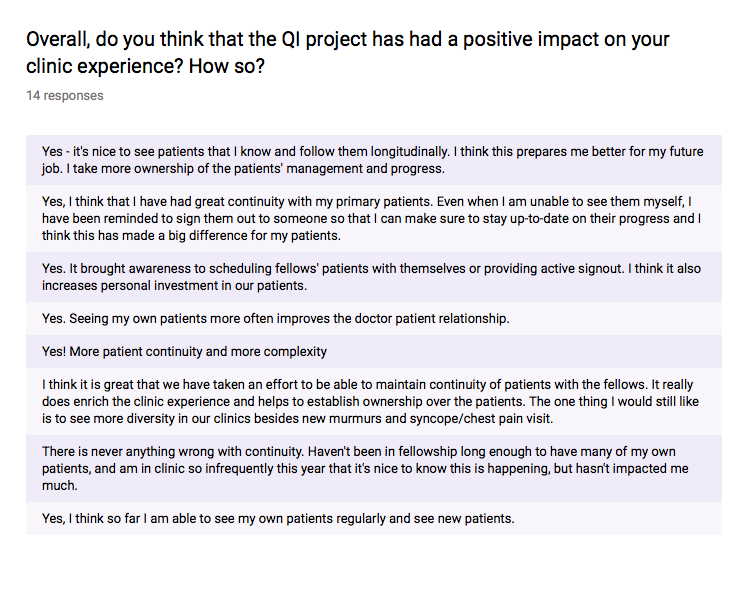

Supplement: Supplementary file 1 [file pqs-5-e306-s001.docx]
